# Adoptive TIL in HPV-negative oral scca

**DOI:** 10.1186/2051-1426-3-S2-P26

**Published:** 2015-11-04

**Authors:** Rom Leidner, Brendan D Curti, Roxanne M Payne, Walter Urba, Keith S Bahjat, Yoshinobu Koguchi, Krista Nelson, Jennifer A Moore, Marka Crittenden, Yedeh Ying, Ashish Patel, Allen Cheng, Tuan G Bui, Traci Hilton, Christopher Paustian, Sachin Puri, Hong Ming Hu, Carlo Bifulco, Zipei Feng, R Bryan Bell, Bernard Fox

**Affiliations:** 1 Earl A. Chiles Research Institute, Providence Cancer Center, Portland, OR, USA; 2 Providence Cancer Center, Providence Portland Medical Center, Portland, OR, USA; 3 Robert W. Franz Cancer Research Center, Portland, OR, USA; 4 The Oregon Clinic, Portland, OR, USA; 5 Head & Neck Institute, Portland, OR, USA; 6 UbiVac, Portland, OR, USA

## Background

A 22 year old male was diagnosed with clinical T2 squamous cell carcinoma of the lateral oral tongue. He underwent partial glossectomy with elective neck dissection of ipsilateral cervical nodes levels I-IV. Pathology showed a 2.1cm primary tumor with clear margins to 5mm, 1.6cm depth of invasion, one of 23 lymph nodes with a 3.5mm focus of involvement at level II, sans extra-capsular extension (pT2 pN1 M0).

## Methods

Through an institutional research tissue protocol, a primary cell line was successfully established. TIL were simultaneously cultured and demonstrated tumor specific IFN-γ production. He completed a course of adjuvant IMRT to 60Gy in the ipsilateral neck and 54Gy in the contralateral neck within 12 weeks of surgery, without complications.

## Results

Three months following completion of radiation, bilateral neck nodal recurrence was noted on scans and confirmed by biopsy (month 6 of disease course, overall). He was treated first line chemotherapy for recurrent disease during months 7 to 10, progressing locally in the neck through two multi-drug regimens (cisplatin/cetuximab/5FU, carboplatin/docetaxel). He then enrolled on a Phase Ib trial of combination anti-PD-1/anti-KIR during months 11 to 14 (BMS CA223-001, nivolumab/lirilumab), again progressing locally in the bilateral neck. At month 15, an individual patient compassionate treatment protocol was approved (FDA IND #16279, PH&S IRB #14-323B), using reduced-intensity Cy/Flu preparative lymphodepletion followed by adoptive transfer of expanded autologous TIL in conjunction with DC-targeted microvessicle DPV-001 intradermal vaccine (DRibbles) and imiquimod adjuvant, with high-dose IL-2 support. Peripheral immune monitoring was performed using whole blood time-course sampling. Multi-spectral immunofluorescence with digital image quantitative immunophenotyping of FFPE longitudinal biopsy specimens was performed using the PerkinElmer Vectra® platform. Data and results analysis will be presented in poster format.

